# Change in Obesity Prevalence across the United States Is Influenced by Recreational and Healthcare Contexts, Food Environments, and Hispanic Populations

**DOI:** 10.1371/journal.pone.0148394

**Published:** 2016-02-05

**Authors:** Candice A. Myers, Tim Slack, Corby K. Martin, Stephanie T. Broyles, Steven B. Heymsfield

**Affiliations:** 1 Pennington Biomedical Research Center, Baton Rouge, Louisiana, 70808, United States of America; 2 Department of Sociology, Louisiana State University, Baton Rouge, Louisiana, 70803, United States of America; University of Rhode Island, UNITED STATES

## Abstract

**Objective:**

To examine change in county-level adult obesity prevalence between 2004 and 2009 and identify associated community characteristics.

**Methods:**

Change in county-level adult (≥20 years) obesity prevalence was calculated for a 5-year period (2004–2009). Community measures of economic, healthcare, recreational, food environment, population structure, and education contexts were also calculated. Regression analysis was used to assess community characteristics associated (p<0.01) with change in adult obesity prevalence.

**Results:**

Mean±SD change in obesity prevalence was 5.1±2.4%. Obesity prevalence decreased in 1.4% (n = 44) and increased in 98% (n = 3,060) of counties from 2004–2009. Results showed that both baseline levels and increases in physically inactive adults were associated with greater increases in obesity prevalence, while baseline levels of and increases in physician density and grocery store/supercenter density were related to smaller increases in obesity rates. Baseline levels of the Hispanic population share were negatively linked to changing obesity levels, while places with greater Hispanic population growth saw greater increases in obesity.

**Conclusions:**

Most counties in the U.S. experienced increases in adult obesity prevalence from 2004 to 2009. Findings suggest that community-based interventions targeting adult obesity need to incorporate a range of community factors, such as levels of physical inactivity, access to physicians, availability of food outlets, and ethnic/racial population composition.

## Introduction

Recent evidence indicates that growth trends in obesity prevalence among adults in the United States (U.S.) may be slowing down.[1] However, despite this potential good news, obesity prevalence remains high in the U.S., especially in comparison to similar countries, and warrants continued monitoring.[1,2] Ecological obesity research has demonstrated the uneven geographic distribution of obesity across the U.S. and identified a range of determinants associated with this geographic variation.[3,4] Specifically, recent research has investigated obesity prevalence across various geographic delineations (i.e., counties and census tracts) and assessed the associated aggregate risk factors (e.g., socioeconomic status [SES]).[5,6] These studies provide an empirical foundation for considering the importance of spatial disparities in obesity prevalence as well as its ecological correlates. However, an important shortcoming of these studies and others is the use of single time point cross-sectional designs.[3,5–8] Such approaches afford only a static snapshot of the geographic distribution of obesity prevalence and community characteristics related to such geographic variation. Insomuch as it is important to monitor trends in overall adult obesity prevalence, it is also important to understand how the geography of adult obesity prevalence has changed in recent years across the U.S.

In the current study, we investigated how county-level adult obesity prevalence changed between 2004 and 2009 and what community characteristics influenced this change. Answering these questions allowed us to describe changing obesity levels in the U.S. and identify those community risk factors most important in affecting the temporal trajectory of obesity levels across the U.S. To our knowledge, this was the first study to investigate change in the geography of obesity in the U.S. and thus provides important information for geographically-targeted obesity intervention efforts.

## Methods

### Dependent Variable

We relied upon county-level obesity estimates from the Center for Disease Control and Prevention (CDC) to create our dependent variable. [9] County-level estimates of diabetes and selected risk factors (e.g., obesity, leisure-time physical inactivity) are model-based and derived from data using the CDC’s Behavioral Risk Factor Surveillance System (BRFSS)[10] and the U.S. Census Bureau’s Population Estimates Program. [11] While the BRFSS currently samples from nearly every county in the nation, small sample sizes prevent the direct calculation of reliable county-specific estimates for most U.S. counties. To overcome this limitation, the CDC has drawn on the aforementioned data to develop county-level obesity prevalence estimates for all U.S. counties using model-based small area estimation techniques. To increase the precision of year-specific county-level estimates, 3 years of BRFSS data are pooled for a given time point. For example, the CDC estimates for 2009 were based on data from 2008, 2009, and 2010, totaling approximately 1.3 million respondents. Validation-studies have compared estimates produced by this modeling technique against direct estimates from counties with large enough sample sizes and have shown little disagreement between the direct and model-based estimates. [12] Those involved in the production of the CDC’s diabetes and associated risk factors estimates have encouraged research that explicitly incorporates spatial effects to describe and account for county-level patterns in these data. [13,14]

Using the CDC county-level obesity estimates, our dependent variable was the percentage-point change in age-adjusted adult obesity prevalence between 2004 and 2009 in the contiguous U.S. (including parishes in Louisiana and independent cities in Virginia; counties in Alaska and Hawaii were excluded). Specifically, we used the estimate for the percent of the adult population (≥ 20 years) that was obese (BMI≥30kg/m^2^) within a county, to calculate the percentage-point difference between the two time periods: Time 2 (2009)–Time 1 (2004).

### Independent Variables

Six dimensions of county-level characteristics were assessed in relation to change in local adult obesity prevalence: economic context, healthcare context, recreational context, food environment, population structure, and education context. These six dimensions were the focus of our analysis given prior work that has cited these factors as important community influences in relation to population health.[5,15,16] We used data available from multiple national sources, including the CDC, U.S. Census Bureau, U.S. Department of Agriculture (USDA), and U.S. Department of Health and Human Services (HSS),to compile a dataset that allowed us to include all counties in the contiguous U.S. and to examine a wide range of relevant predictors of obesity prevalence. For the CDC’s county-level estimates, 2004 (Time 1) was the earliest year for which age-adjusted adult obesity is available. However, 2000 was the best available earliest year (Time 1) for the majority of independent variables. We measured the local *economic context* as: 1) percent of the population living in poverty; 2) percent of the labor force unemployed; and 3) residential segregation of the poor from the non-poor. Data for these measures were obtained from the 2000 Decennial U.S. Census and 2005–2009 American Community Survey (ACS) 5-year Estimates. Cross-sectional analyses have indicated that these economic indicators are associated with chronic disease prevalence across U.S. counties. [5,13,16] We sought to understand how these economic measures shaped changing levels of obesity prevalence.

Local healthcare context was quantified as: 4) percent of the population without health insurance; 5) number of physicians per 1,000 people; and 6) number of outpatient hospital visits per 1,000 people. Health insurance data were obtained from the U.S. Census Bureau’s Small Area Health Insurance Estimates. Both physician and outpatient visits data were from the HHS Area Resource File (ARF). While it has been demonstrated that these measures are important indicators of community health in single time point cross-sectional analyses, we aimed to evaluate how the healthcare context of places influenced change in obesity prevalence.[15,16]

Community recreational context was measured as: 7) percent of adults who were physically inactive and 8) number of fitness and recreation centers per 1,000 people. Physical inactivity data for adults were obtained from the CDC’s Diabetes Interactive Atlas, County-Level Estimates of Leisure-Time Physical Activity. The number of fitness and recreation centers, as identified by the North American Industry Classification System (NAICS) code 713940, per 1,000 people was drawn from the USDA’s Economic Research Service (ERS) Food Environment Atlas, which used County Business Patterns (CBP) and Population Estimates (U.S. Census Bureau) data. Given evidence demonstrating the effect of aggregate levels of physical inactivity and access to recreational and fitness facilities during single cross-sections, we sought to understand how these community attributes impacted obesity prevalence change.[17,18]

The food environment was measured as: 9) number of grocery stores (NAICS code 445110) and supercenters (NAICS 452910) per 1,000 people and 10) number of fast food restaurants (NAICS code 722211) per 1,000 people. Both variables were drawn from the USDA’s ERS Food Environment Atlas. Research has demonstrated that the availability of food outlets is associated with obesity rates.[19,20] Our intent was to examine how access to these food outlets was related to change obesity levels.

Change in population structure was assessed as: 11) percent of families with children headed by single women; and 12) percent of the population 65 years and older, 13) percent of the population African American, and 14) percent of the population Hispanic. These variables were obtained from the U.S. Census Bureau’s 2000 Decennial Census and 2005–2009 ACS 5-year Estimates. Single time point cross-sectional evidence has shown the significance of population structure measures in relation to obesity prevalence.[5,16] However, we were interested in determining how these measures influenced change in obesity prevalence.

Education context was captured by 15) percent of adults without a high school degree or equivalent. This variable was from the 2000 Decennial U.S. Census and 2005–2009 ACS 5-year Estimates. While others have shown the importance of educations levels in relation to obesity prevalence, we aimed to assess the relationship between human capital levels and changing obesity prevalence across U.S. counties.[5,15]

## Statistical Analysis

We first used descriptive statistics and mapping to examine county-level variation in change in adult obesity prevalence. We next regressed a weighted least squares (WLS) regression model with all independent variables measured as 1) baseline values (Time 1 [circa 2000]) and 2) difference scores (Time 2 [circa 2009] minus Time 1 [circa 2000]). This modeling strategy allowed us to investigate: 1) how baseline conditions influenced change in adult obesity prevalence; and 2) how change in adult obesity prevalence was associated with concurrent changes in community characteristics. By measuring covariates at an earlier time point (Time 1), we detected how baseline community features influenced the general trend or direction of change in obesity prevalence.[21] Measuring covariates as difference scores allowed us to assess how changing community features have impacted the trajectory or path of obesity prevalence.[21] This modeling strategy helped to rule out reciprocal effects and controlled for time invariant factors, thus coming closer to isolating causal linkages between change in adult obesity prevalence and community characteristics than allowed by single time point cross-sectional studies.

We weighted our regression models by the total county population size in 2000. We did so because ordinary least squares regression weights all counties equally such that a percentage-point change in a county with a 1,000 residents exerts the same influence in the model as a percentage-point change in a county with 1,000,000 residents. We also included baseline adult obesity prevalence (2004) to control for potential ceiling effects in the dependent variable. To account for multiple comparisons in the regression models, we reported significant findings using an alpha level of p<0.01.

Model diagnostics did not indicate any issue with collinearity among predictors, baseline and change, in our regression model (variance inflation factors <10).[22] Based upon previous ecological analyses [5,16], we also took steps to address two related issues specific to county-level regression analyses: state-level effects and spatial autocorrelation. Because counties are nested in states, which are comprised of different numbers of counties, unmeasured variables that are county-invariant within a state can bias county-level regression estimates. We controlled for state-fixed effects to account for potential state-based bias. Beyond state-specific effects, local conditions in a county can also be a function of proximity to neighboring counties. Known as spatial autocorrelation, this too violates regression assumptions and can potentially bias regression estimates. To diagnose the degree to which spatial autocorrelation existed in our data we conducted a Local Indicators of Spatial Association (LISA) analysis and examined the Moran’s I statistic, using a ‘queen’ weights matrix to define each county’s neighboring counties, to discern if spatial autocorrelation was present in our data.[23–26] We relied upon the statistical package GeoDa 1.6.7 to carry out the spatial diagnostics.[27] All additional analyses were carried out using IBM SPSS Statistics Version 20. Mapping was done using ArcGIS 10.2.

## Results

### Descriptives and Mapping: Change in Adult Obesity Prevalence

**Fig 1:**
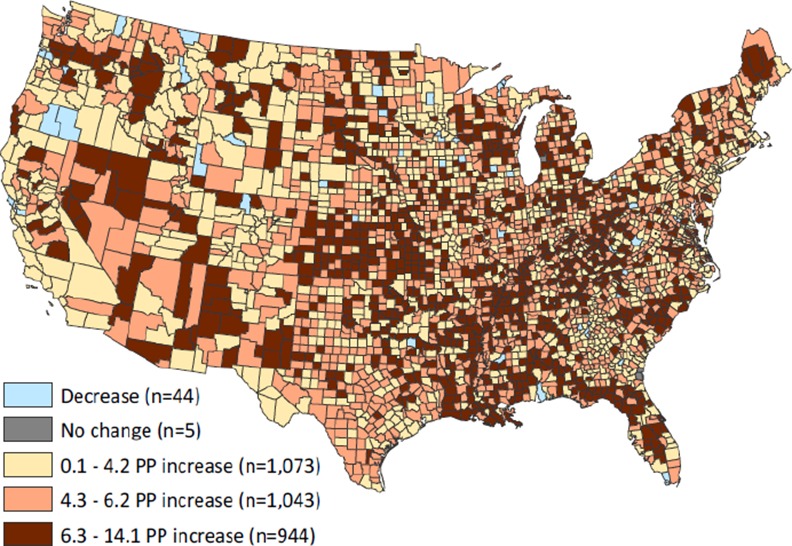
Map of change in county-level adult obesity prevalence, 2004–2009.

County-level adult obesity increased an average of 5.1 percentage points between 2004 and 2009, ranging from a decrease of 3.7 to an increase of 14.1 (Table 1). The geographic distribution of change in county-level adult obesity is illustrated in Fig 1. Between 2004 and 2009, obesity prevalence decreased in 1.4% (n = 44) and increased in 98% (n = 3 060) of counties, while 0.2% (n = 5) had no change. A key point illustrated by this map was the lack of large and distinct regional clusters of obesity change. This stands in stark contrast to the pronounced regional concentrations of obesity prevalence that have been demonstrated in single time point cross-sectional studies.[3,5,16] Rather, spatial clusters of obesity change were generally small with no particular spatial patterns concentrated in certain states or regions (e.g., the South). Results for the LISA analysis, which revealed only modest positive spatial autocorrelation (i.e., geographic clustering of counties with like values, Moran’s I = 0.16; p<0.05) in county-level adult obesity change are available in S1 Fig.

**Table 1:** Descriptive Statistics.

	Mean	S.D.	Min.	Max.
**Dependent variable**				
PP Δ adults obese, 2004–2009	5.1	2.4	-3.7	14.1
**Independent variables**				
Percent of adults obese, 2004	25.2	3.3	12.3	37.9
*Economic Context*				
Percent of pop. poor, 2000	14.2	6.5	0.0	14.2
PP Δ poverty, 2000–2009	1.3	3.3	-20.7	37.1
Percent of labor force unemployed, 2000	3.4	1.4	0.0	28.0
PP Δ labor force unemployed, 2000–2009	0.7	1.6	-24.4	13.7
Poor/non-poor segregation, 2000	15.9	10.7	0.0	51.3
PP Δ poor/non-poor segregation, 2000–2009	2.9	6.2	-24.1	50
*Healthcare Context*				
Percent of pop. uninsured, 2000	14.7	5.0	0.0	38.0
PP Δ uninsured, 2000–2009	3.6	3.4	-16.1	20.3
Number of physicians/1,000 pop., 2000	1.4	1.5	0.0	21.4
Δ physicians/1,000 pop., 2000–2009	0.1	0.5	-8.1	10.3
Number of outpatient visits/per 1,000 pop., 2000	1,895.7	2,251.2	0.0	41,206.5
Δ outpatient visits/per 1,000 pop., 2000–2009	535.1	2,241.3	-22,798.3	31,139.8
*Recreational Context*				
Percent of adults physically inactive 2004	25.3	5.2	9.9	41.8
PP Δ adults physically inactive 2004–2009	1.6	3.1	-10.2	12.9
Number of recreation facilities/1,000 pop., 2000	0.1	0.3	0.0	1.2
Δ recreation facilities/1,000 pop., 2000–2009	0.01	0.1	-1.2	0.7
*Food Environment*				
Number of grocery stores & supercenters/1,000 pop., 2000	0.3	0.2	0.0	2.9
Δ grocery stores & supercenters/1,000 pop., 2000–2009	-0.1	0.2	-1.8	2.1
Number of fast food restaurants/1,000 pop., 2000	0.5	0.3	0.0	5.4
Δ fast food restaurants/1,000 pop., 2000–2009	0.03	0.2	-2.7	1.8
*Population Structure*				
Percent of families headed by single mothers, 2000	8.7	3.4	0.0	28.8
PP Δ families headed by single mothers, 2000–2009	1.0	2.2	-15.4	13.4
Percent of pop. aged 65 and older, 2000	14.8	4.1	1.8	34.7
PP Δ aged 65 years and older, 2000–2009	0.5	1.5	-14.9	17.7
Percent of pop. African American, 2000	8.7	14.5	0.0	86.1
PP Δ African American, 2000–2009	0.1	1.4	-12.0	18.4
Percent of pop. Hispanic, 2000	6.2	12.1	0.0	98.1
PP Δ Hispanic, 2000–2009	1.4	2.0	-17.9	22.7
*Human Capital*				
Percent of adults less than high school, 2000	22.7	8.7	3.0	65.3
PP Δ adults less than high school, 2000–2009	-5.6	2.9	-19.0	9.4

*Notes*: PP Δ = percentage-point change, Δ = change, pop. = population. PP Δ variables are difference measures: Time 2- Time 1. Obesity and physical inactivity data are from the CDC County-level Estimates of Obesity and Leisure-Time Physical Inactivity. Uninsured data are from the U.S. Census Bureau, Small Area Health Insurance Estimates. Physician data are from the Area Resource File, via the Dept. of Health and Human Services. Recreation facilities, grocery stores and supercenters, and fast food restaurant data are from the USDA Economic Research Service Food Environment Atlas and the U.S. Census Bureau County Business Patterns. All other data is obtained from the U.S. Census Bureau: 2005–2009 American Community Survey 5-year Estimates and the 2000 Decennial U.S. Census. N = 3,109.

### Regression: Change in Adult Obesity Prevalence

**Fig 2:**
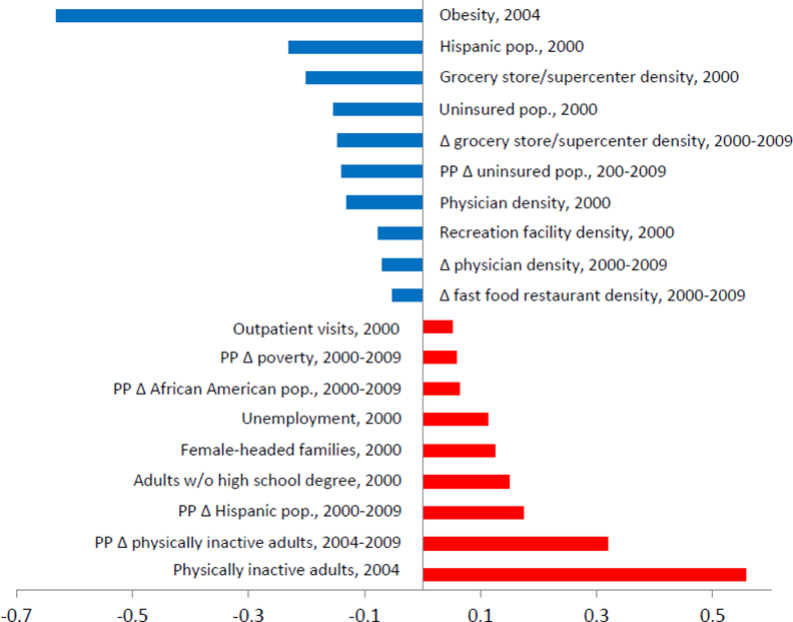
Significant (p<0.01) standardized coefficients from WLS regression model of change in county-level adult obesity prevalence, 2004–2009. Blue and red bars represent significant negative and positive correlations with change in county-level adult obesity prevalence, respectively. Intercept = 6.684 [5.432, 7.936]*. Adjusted R^2^ = 0.397.

In **Fig 2** we provide a graphical image of the significant (p<0.01) standardized regression coefficients to provide a useful effect size measure that indicates the relative strength of each predictor included in the model (unstandardized coefficients are available in S1 Table). Because a slight signal of spatial autocorrelation was shown in our data, we carried out a supplementary regression analysis that included a spatial lag term controlling for these effects in the model. The spatial lag was calculated as the average value of the dependent variable (i.e., percentage-point change in adult obesity prevalence) among a county’s neighbors (S2 Table). However, because the substantive results and conclusions are comparable between the primary and supplementary analyses, we present results from the WLS model with state-fixed effects only for the sake of parsimony.

Five baseline conditions showed significant positive associations with obesity change, including unemployment, outpatient visits, physically inactive adults, female-headed families, and adults without a high school degree. Conversely, five baseline parameters showed significant negative relationships with obesity change, including uninsured populations, physician density, recreation facility density, grocery store/ supercenter density, and Hispanic populations. These results were robust to the inclusion of state-fixed effects.

Standardized coefficients (β) from the model indicated that the proportion of adults within counties that were physically inactive in 2004 (b = 0.268; β = 0.557; 95% CI = 0.227 0.310) had the strongest positive influence on change in adult obesity prevalence among all baseline county covariates. That is, counties witnessed greater increases in obesity prevalence from 2004 to 2009 where there were larger shares of physically inactive adults in 2004. In contrast, aside from the control for baseline obesity prevalence (b = -0.389; β = -0.632; 95% CI = -0.432, -0.347), Hispanic populations (b = -0.033; β = -0.230; 95% CI = -0.045, -0.021) had the strongest negative influence on change in adult obesity prevalence among all baseline county covariates. In other words, those counties that had comparatively larger proportions of Hispanic populations in 2000 experienced smaller increases in obesity prevalence during the 5 year period.

A number of changes in community features also influenced change in obesity prevalence. Change in poverty rates, physically inactive adults, African American populations, and Hispanic populations were positively related to change in obesity prevalence. Difference measures for uninsured populations, physician density, grocery store/supercenter density, and fast food restaurant density were negatively associated with change in obesity prevalence. Again, all statistical findings were robust to the inclusion of state-fixed effects.

Similar to baseline covariates, change in physically inactive adults (b = 0.294; β = 0.319; 95% CI = 0.259, 0.329) was the strongest positive predictor of changing levels of obesity prevalence among all difference measures. From 2004 to 2009, those counties that saw increases in the amount of physically inactive adults also saw increases in adult obesity prevalence. Aside from the control for obesity prevalence at baseline, change in grocery store/supercenter density (b = -4.340; β = -0.147; 95% CI = -5.496, -3.184) was the strongest negative predictor of obesity prevalence change among all difference parameters. That is, lesser increases in adult obesity prevalence were seen in counties with increases in the availability of grocery stores/supercenters.

Across both baseline and difference covariates in the model, four community features were significant. These included physically inactive adults, which were positive covariates, physician density and grocery store/supercenter density, which were negative covariates, and the Hispanic population share, which was a negative baseline covariate and positive change covariate. These particular county characteristics, physical inactivity, physician availability, access to grocery stores/supercenters, and Hispanic populations, played a twofold role in influencing both the trend and overall trajectory of how obesity prevalence changed from 2004 to 2009 in the U.S.

## Discussion

The study examined change in adult obesity across U.S. counties from 2004 to 2009 and identified community-level determinants associated with this outcome. We found that most counties experienced increases in adult obesity during the last half of the previous decade. Further, while significant clusters of low and high change counties were found, these areas did not demonstrate distinct regional patterns. This stands in marked contrast to the significant and pronounced regional concentrations of obesity prevalence demonstrated in cross-sectional studies examining single points in time. Taken together, our findings move beyond cross-sectional snapshots and demonstrate the temporality of adult obesity prevalence across the U.S.

Regarding significant baseline measures, counties with larger proportions of unemployed labor force participants, female-headed households, and adults without a high school education experienced greater increases in obesity prevalence. These three measures speak to SES within counties and indicate that low community-level SES is associated with growth in obesity prevalence. Counties with greater outpatient visits also experienced greater increases in obesity prevalence. This relationship perhaps signals a greater need for medical care necessitated by the range of comorbid health problems associated with obesity. Conversely, greater density of recreation facilities was associated with lessened increases in county-level obesity prevalence, providing further support for the relationship between access to recreational facilities and lesser obesity rates shown elsewhere.[28]

Examining difference measures, change in fast food restaurant density was negatively associated with change in obesity prevalence across U.S. counties. That is, for those counties with increases in fast food restaurants, obesity prevalence increased less. While there is evidence that access to fast food restaurants is associated with higher obesity rates,[20] ecological research has also shown negative relationships between fast food restaurant density and obesity rates [28,29]. Essentially, the relationship between the food environment and obesity is not fully elucidated, with many contradictory associations between food outlets and chronic disease prevalence found in the literature.[30] Increases in African American populations were associated with increases in adult obesity prevalence. This particular relationship has been shown in other cross-sectional analyses of county-level obesity rates, [5,28] with the effect being particularly pronounced in the South.[16] Similarly, increases in county poverty rates were also associated with increases in adult obesity prevalence. Other ecological studies have found comparable relationships between poverty and adult obesity in both single cross-sectional and change analyses.[17,28] Last, we note that both baseline and change measures for uninsured populations were negatively associated with change in adult obesity prevalence. This is to say that communities that had both larger proportions of and increases in the population without health insurance saw smaller increases in obesity prevalence. Other cross-sectional research has shown a negative relationship between uninsured populations and obesity prevalence, however this association was demonstrated to be driven by conditions specific to the West Census Bureau region of the U.S. (i.e., Colorado to California).[16] We point out that our analysis was ecological and did not capture actual utilization of health insurance and corresponding medical services, but rather health insurance coverage at the population level.

Results revealed a number of community characteristics that were particularly notable for their linkages to change in adult obesity prevalence. Counties with both higher baseline levels of physically inactive adults and growth in physically inactive adults witnessed greater increases in adult obesity prevalence. These findings are intuitive given the important relationship between physical activity and obesity. Greater physician density at both baseline and during the change period was associated with lesser increases in obesity prevalence, suggesting that physicians serve as an important healthcare resource within counties. Availability of grocery stores/supercenters both at the beginning and increases during the assessment period were associated with less growth in obesity prevalence. Other studies have also shown that obesity prevalence is lower in areas with greater access to supermarkets.[20,31] Turning to Hispanic populations, the results showed countervailing effects, with baseline population levels shown to have a negative effect on change in obesity prevalence, while places with growing Hispanic populations were linked to greater increases in obesity levels. Previous cross-sectional research has found a significantly negative association between county-level Hispanic populations and lower chronic disease prevalence.[13] Moreover, while the greatest concentrations of Hispanic populations continue to be in the Southwest and West (regions with relatively low obesity prevalence), the places where Hispanic populations are growing most rapidly are in the Southeast and Midwest (regions with relatively high obesity prevalence).[32] It is worth noting research that has documented the influence of acculturation (i.e., the adoption of dietary and physical activity habits typical in the U.S.) on the health status of U.S. Hispanics, [33–35] However, our data do not allow us to speak to acculturation issues, such as nativity (foreign-born versus U.S.-born) or Hispanic origin (e.g., Mexican-origin versus Spanish-origin), that may be relevant. Taken together, these results point to the importance of both baseline and changing community conditions in shaping how obesity prevalence changed from 2004 to 2009. Finally, adult obesity prevalence at baseline (2004) showed a significant negative effect on change in adult obesity prevalence. This indicates a potential ‘ceiling effect’ where obesity increased most in counties that were not traditionally high obesity places.

This study has several potential shortcomings. As noted in a previous report,[36] there are limitations associated with the CDC’s county-level estimates. These are model-based estimates derived from the BRFSS, which obtains health data by self-report and via a random sample of landline telephones. Therefore, biases associated with the modeling strategy, underestimates of body weight and overestimates of height, and the exclusion of cellphone-only households impose limitations in terms of generalizability.[4] Population health census data would be invaluable for obesity research, policy, and intervention, but it currently does not exist in the U.S. Additionally, our results may be influenced by the slight temporal mismatch between change in county-level adult obesity prevalence (2004 to 2009) and change in local-level factors (2000/2004 to 2009). Optimally, we would have examined change in county-level adult obesity prevalence for the entire previous decade, 2000 to 2009, but the lack of CDC estimates at the beginning of the decade prohibited this. Our findings are also vulnerable to both the modifiable areal unit problem (MAUP) and the uncertain geographic context problem (UGCoP), which are limitations intrinsic to most ecological analyses in that the spatial units of analysis can change over time (e.g., county boundaries) and were created for purposes other than that under study (e.g., counties are government administrative units not health districts), and that ultimately the proper scale is not known (e.g., obesity might be better measured at lower levels like neighborhoods or higher levels like states).[37,38]

This study is the first to our knowledge that addresses change in county-level adult obesity prevalence across the contiguous U.S. Our results highlight the community-based forces associated with increasing obesity prevalence. One implication to be drawn from this study is that no single aspect of community life is solely responsible for obesity levels; rather, a range of varying factors from multiple contexts were implicated in the temporal trajectory of obesity prevalence. This highlights the need to consider community-based intervention efforts that address a spectrum of factors in concert in order for obesity to be effectively reduced.[39] Second, in contrast to pronounced regional obesity regimes demonstrated for obesity prevalence, geographic disparities in obesity change were not nearly as evident. The implication here is that while obesity levels may be a more pressing population health concern in particular regions of the country, rising obesity prevalence is truly a widespread national concern.

## Supporting Information

S1 Dataset Obesity Change 2004 2009 Baseline and Difference Reduced. (XLSX)

S1 Fig Local Indicators of Spatial Association (LISA) map of significant clusters of change in county-level adult obesity prevalence, 2004–2009. Moran’s I = 0.16; p<0.05. Blue shaded counties are core members of geographic clusters with significantly (p<0.05) lower change in adult obesity prevalence than would be expected at random (Low-low). A total of 7% (n = 210) of U.S. counties were identified as core members of significant low obesity change clusters. Red shaded counties are core members of geographic clusters with significantly (p<0.05) higher change in adult obesity prevalence (High-high). A total of 7% (n = 228) of U.S. counties were shown to be core counties of clusters with significant high obesity change.
(TIF)

S1 Table WLS regression model of change in county-level adult obesity prevalence, 2004–2009. (DOCX)

S2 Table WLS regression model of change in county-level adult obesity prevalence, 2004–2009. (DOCX)
