# Supplementary figures and images for: Change in Obesity Prevalence across the United States Is Influenced by Recreational and Healthcare Contexts, Food Environments, and Hispanic Populations
Source: PLoS One. 2016 Feb 5;11(2):e0148394. doi: 10.1371/journal.pone.0148394 (PMC4743954; doi:10.1371/journal.pone.0148394)

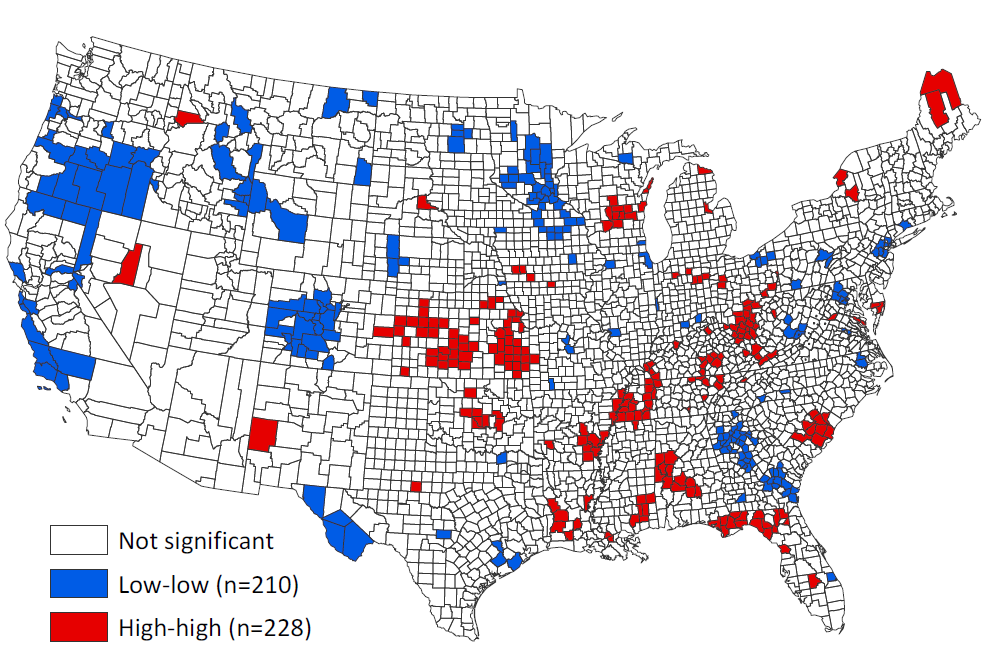

Supplement: S1 Fig — Moran’s I = 0.16; p<0.05. Blue shaded counties are core members of geographic clusters with significantly (p<0.05) lower change in adult obesity prevalence than would be expected at random (Low-low). A total of 7% (n = 210) of U.S. counties were identified as core members of significant low obesity change clusters. Red shaded counties are core members of geographic clusters with significantly (p<0.05) higher change in adult obesity prevalence (High-high). A total of 7% (n = 228) of U.S. counties were shown to be core counties of clusters with significant high obesity change. (TIF) [file pone.0148394.s002.tif]
