# Supplementary material for: Change in Obesity Prevalence across the United States Is Influenced by Recreational and Healthcare Contexts, Food Environments, and Hispanic Populations
Source: PLoS One. 2016 Feb 5;11(2):e0148394. doi: 10.1371/journal.pone.0148394 (PMC4743954; doi:10.1371/journal.pone.0148394)
Supplement: S1 Table — (DOCX) [file pone.0148394.s003.docx]

| **Table S1. WLS regression model of change in county-level adult obesity prevalence, 2004-2009** | |
| --- | --- |
|  |  |
| **Variables** | b (β) [95% CI] |
| *Economic Context* |  |
| Percent of pop. poor, 2000 | 0.037 (0.094) [0.000, 0.074] |
| PP ∆ poverty, 2000-2009 | 0.060 (0.059) [0.015, 0.104]* |
| Percent of labor force unemployed, 2000 | 0.207 (0.112) [0.084, 0.331]* |
| PP ∆ labor force unemployed, 2000-2009 | 0.067 (0.033) [-0.027, 0.161] |
| Poor/non-poor segregation, 2000 | -0.015 (-0.067) [-0.026, -0.003] |
| PP ∆ poor/non-poor segregation, 2000-2009 | -0.006 (-0.011) [-0.024, 0.012] |
| *Healthcare Context* |  |
| Percent of pop. uninsured, 2000 | -0.073 (-0.153) [-0.127, -0.019]* |
| PP ∆ uninsured, 2000-2009 | -0.086 (-0.140) [-0.126, -0.047]* |
| Number of physicians/1,000 pop., 2000 | -0.151 (-0.130) [-0.207, -0.095]* |
| ∆ physicians/1,000 pop., 2000-2009 | -0.425 (-0.069) [-0.641, -0.209]* |
| Number of outpatient visits/per 1,000 pop., 2000 | 0.071 (0.051) [0.022, 0.120]* |
| ∆ outpatient visits/per 1,000 pop., 2000-2009 | 0.057 (0.036) [0.010, 0.104] |
| *Recreational Context* |  |
| Percent of adults physically inactive 2004 | 0.268 (0.557) [0.227, 0.310]* |
| PP ∆ adults physically inactive 2004-2009 | 0.294 (0.319) [0.259, 0.329]* |
| Number of recreation facilities/1,000 pop., 2000 | -3.953 (-0.077) [-6.245, -1.661]* |
| ∆ recreation facilities/1,000 pop., 2000-2009 | -2.027 (-0.033) [-4.183, 0.129] |
| *Food Environment* |  |
| Number of grocery stores & supercenters/1,000 pop., 2000 | -4.184 (-0.200) [-5.123, -3.184]* |
| ∆ grocery stores & supercenters/1,000 pop., 2000-2009 | -4.340 (-0.147) [-5.496, -3.184]* |
| Number of fast food restaurants/1,000 pop., 2000 | 0.257 (0.020) [-0.288, 0.803] |
| ∆ fast food restaurants/1,000 pop., 2000-2009 | -1.041 (-0.051) [-1.760, -0.321]* |
| *Population Structure* |  |
| Percent of families headed by single mothers, 2000 | 0.078 (0.125) [0.029, 0.127]* |
| PP ∆ families headed by single mothers, 2000-2009 | 0.029 (0.016) [-0.031, 0.089] |
| Percent of pop. aged 65 and older, 2000 | 0.006 (0.010) [-0.022, 0.034] |
| PP ∆ aged 65 years and older, 2000-2009 | 0.080 (0.033) [-0.012, 0.171] |
| Percent of pop. African American, 2000 | -0.014 (-0.082) [-0.026, -0.002] |
| PP ∆ African American, 2000-2009 | 0.101 (0.063) [0.044, 0.158]* |
| **Table S1. Cont’d.** |  |
| Percent of pop. Hispanic, 2000 | -0.033 (-0.230) [-0.045, -0.021]* |
| PP ∆ Hispanic, 2000-2009 | 0.204 (0.175) [0.147, 0.261]* |
| *Human Capital* |  |
| Percent of adults less than high school, 2000 | 0.044 (0.149) [0.014, 0.073]* |
| PP ∆ adults less than high school, 2000-2009 | 0.048 (0.047) [-0.011, 0.106] |
| *Controls* |  |
| Obesity, 2004 | -0.389 (-0.632) [-0.432, -0.347]* |
| Intercept | 6.684 (----) [5.432, 7.936]* |
| Adjusted R^2^ | 0.397 |

*Notes*: PP ∆=percentage-point change, ∆=change, pop.=population. *b* signifies unstandardized regression coefficients. *β* signifies standardized regression coefficients. Model controls for state fixed effects and is weighted by county total population size, 2000. ∆ outpatient visits/1 000 pop., 2000-2009 coefficient and 95% C.I. multiplied by 1 000. N=3 109. *p<.01.
