# Supplementary material for: Change in Obesity Prevalence across the United States Is Influenced by Recreational and Healthcare Contexts, Food Environments, and Hispanic Populations
Source: PLoS One. 2016 Feb 5;11(2):e0148394. doi: 10.1371/journal.pone.0148394 (PMC4743954; doi:10.1371/journal.pone.0148394)
Supplement: S2 Table — (DOCX) [file pone.0148394.s004.docx]

| **Table S2. WLS regression model of change in county-level adult obesity prevalence, 2004-2009** | |
| --- | --- |
|  |  |
| **Variables** | b (β) [95% CI] |
| *Economic Context* |  |
| Percent of pop. poor, 2000 | 0.030 (0.077) [-0.007, 0.068] |
| PP ∆ poverty, 2000-2009 | 0.057 (0.056) [0.012, 0.101] |
| Percent of labor force unemployed, 2000 | 0.215 (0.116) [0.092, 0.338]* |
| PP ∆ labor force unemployed, 2000-2009 | 0.071 (0.035) [-0.023, 0.165] |
| Poor/non-poor segregation, 2000 | -0.006 (-0.011) [-0.027, -0.004]* |
| PP ∆ poor/non-poor segregation, 2000-2009 | -0.006 (-0.011) [-0.024, 0.012] |
| *Healthcare Context* |  |
| Percent of pop. uninsured, 2000 | -0.073 (-0.155) [-0.127, -0.019]* |
| PP ∆ uninsured, 2000-2009 | -0.083 (-0.134) [-0.122, -0.043]* |
| Number of physicians/1,000 pop., 2000 | -0.147 (-0.127) [-0.203, -0.092]* |
| ∆ physicians/1,000 pop., 2000-2009 | -0.410 (-0.066) [-0.625, -0.194]* |
| Number of outpatient visits/per 1,000 pop., 2000 | 0.073 (0.052) [0.024, 0.122]* |
| ∆ outpatient visits/per 1,000 pop., 2000-2009 | 0.052 (0.032) [0.004, 0.099] |
| *Recreational Context* |  |
| Percent of adults physically inactive 2004 | 0.273 (0.568) [0.232, 0.315]* |
| PP ∆ adults physically inactive 2004-2009 | 0.294 (0.319) [0.260, 0.329]* |
| Number of recreation facilities/1,000 pop., 2000 | -3.887 (-0.076) [-6.173, -1.600]* |
| ∆ recreation facilities/1,000 pop., 2000-2009 | -2.037 (-0.033) [-4.188, 0.114] |
| *Food Environment* |  |
| Number of grocery stores & supercenters/1,000 pop., 2000 | -4.143 (-0.198) [-5.080, -3.206]* |
| ∆ grocery stores & supercenters/1,000 pop., 2000-2009 | -4.261 (-0.145) [-5.415, -3.107]* |
| Number of fast food restaurants/1,000 pop., 2000 | 0.131 (0.010) [-0.418, 0.679] |
| ∆ fast food restaurants/1,000 pop., 2000-2009 | -1.047 (-0.052) [-1.765, -0.329]* |
| *Population Structure* |  |
| Percent of families headed by single mothers, 2000 | 0.081 (0.131) [0.032, 0.131]* |
| PP ∆ families headed by single mothers, 2000-2009 | 0.025 (0.014) [-0.035, 0.084] |
| Percent of pop. aged 65 and older, 2000 | 0.009 (0.014) [-0.020, 0.037] |
| PP ∆ aged 65 years and older, 2000-2009 | 0.092 (0.038) [0.000, 0.184] |
| Percent of pop. African American, 2000 | -0.011 (-0.068) [-0.023, 0.001] |
| PP ∆ African American, 2000-2009 | 0.104 (0.064) [0.047, 0.161]* |
| **Table S2. Cont’d.** |  |
| Percent of pop. Hispanic, 2000 | -0.029 (-0.204) [-0.042, -0.017]* |
| PP ∆ Hispanic, 2000-2009 | 0.206 (0.177) [0.149, 0.262]* |
| *Human Capital* |  |
| Percent of adults less than high school, 2000 | 0.042 (0.143) [0.013, 0.072]* |
| PP ∆ adults less than high school, 2000-2009 | 0.046 (0.045) [-0.013, 0.104] |
| *Controls* |  |
| Obesity, 2004 | -0.406 (-0.659) [-0.449, -0.363]* |
| ***Spatial lag*** | ***0.122 (0.078) [0.060, 0.185]**** |
| Intercept | 6.466 (----) [5.211, 7.720]* |
| Adjusted R^2^ | 0.400 |

*Notes*: PP ∆=percentage-point change, ∆=change, pop.=population. *b* signifies unstandardized regression coefficients. *β* signifies standardized regression coefficients. Model controls for state fixed effects and is weighted by county total population size, 2000. ∆ outpatient visits/1 000 pop., 2000-2009 coefficient and 95% C.I. multiplied by 1 000. N=3 109. *p<.01.
